# Social Behavior and Group Formation in Male Asian Elephants (*Elephas maximus*): The Effects of Age and Musth in Wild and Zoo-Housed Animals

**DOI:** 10.3390/ani12091215

**Published:** 2022-05-08

**Authors:** Chase A. LaDue, Rajnish P. G. Vandercone, Wendy K. Kiso, Elizabeth W. Freeman

**Affiliations:** 1 Department of Environmental Science and Policy, George Mason University, Fairfax, VA 22030, USA; 2 Department of Biological Sciences, Rajarata University of Sri Lanka, Mihintale 50300, Sri Lanka; vandercone@gmail.com; 3 White Oak Conservation Foundation, Yulee, FL 32097, USA; wkiso@white-oak.org; 4 School of Integrative Studies, George Mason University, Fairfax, VA 22030, USA; efreeman@gmu.edu

**Keywords:** age, all-male groups, Asian elephant, conspecifics, intrasexual competition, mate choice, mixed-sex groups, musth, sexual selection, Sri Lanka

## Abstract

**Simple Summary:**

The conservation of wild and zoo-housed Asian elephants partly depends on developing our understanding of male elephant social behavior. Once thought to be solitary, we now know that male elephants can display rich social exchanges with both males and females. However, these interactions are expected to change with age and around the sexually active state of “musth.” We used behavioral data from wild and zoo-housed elephant populations to investigate how age, musth, and environmental factors influence how male elephants socialize and associate with group members. In Sri Lanka, only male elephants of older age (>20 years) exhibited signs of musth, but even some of the youngest zoo-housed elephants underwent musth (as young as 11 years). Additionally, we found that age and musth status predicted whether a wild male elephant associated with females, other males or was alone, as well as the number of conspecifics (males and females) in the same group with which a male was observed. Finally, rates of aggression, prosocial behavior (affiliative behaviors that promote positive social bonds between elephants), and submissive behavior exhibited by wild and zoo-housed male elephants were associated with age, musth status, the number of elephants presented, and group type (all-male or mixed sex). These results provide motivation for future studies of social behavior in male Asian elephants, as they will contribute to the reproduction and conservation of this endangered species.

**Abstract:**

Asian elephants are endangered, and the long-term viability of the species depends on integrative approaches to address the sustainability of *in-situ* and *ex-situ* populations. Growing evidence shows that male elephants exhibit extensive and flexible social behavior that rivals the complexity of that of females. Male elephant sociality is expected to change dramatically around the unique sexual state of musth. However, data related to male Asian elephant sociality is lacking. Here, we conducted complementary observations in Wasgamuwa National Park, Sri Lanka, and North American zoos of male Asian elephant social behavior. Age and musth status, along with other factors, were associated with variation in social behavior and group formation of males. In wild male elephants, both musth status and age impacted elephant associations within all-male and mixed-sex groups: non-musth elephants were generally sighted less often in mixed-sex groups as they aged, while the inverse occurred with musth elephants. Musth status interacted with age to predict the number of conspecifics with which a wild male elephant associated: younger males were observed with more females during non-musth (but the opposite was true during musth), and male elephants between 20 and 30 years were observed with the highest number of male conspecifics except during musth. Finally, we found variation in aggression, prosocial behavior, and submissive behavior was influenced by intrinsic (age and musth status) and extrinsic factors (group size and type) in similar ways in both populations; prosocial behavior was most common and was influenced by the number of conspecifics present (both populations), and age, group type, and musth status (zoo population), while aggression was rare, especially among older elephants. We suggest that longitudinal studies of this threatened species will be particularly helpful to promote the reproduction and conservation of Asian elephants in *in-situ* and *ex-situ* environments.

## 1. Introduction

Asian elephants (*Elephas maximus*) exhibit polygynous mating strategies, whereby males compete to secure as many mating opportunities as they can afford [1,2,3]. As such, competition among and between males for access to receptive females can be high. Females are philopatric and typically remain in semi-stable natal groups with female relatives and their offspring [4,5], and males disperse in early adulthood to form fewer permanent all-male groups or to move solitarily [6,7,8]. Therefore, to gain access to estrous females, male elephants announce their intent to reproduce through musth. Musth is a reproductive condition that occurs regularly but asynchronously among males [9,10], and it is initiated by a surge in serum androgens that induce a suite of behavioral and physiological changes [11,12,13,14,15]. Musth is also characterized by various signals of multiple sensory modalities (chemical, visual, and/or acoustic components) that apparently promote inter- and intrasexual social interactions [16]. While the social correlates and consequences of musth in African savanna elephants (*Loxodonta africana*) have been well-described [17,18], there is still much to be learned about musth and social behavior in Asian elephants. Further, understanding species-specific drivers of reproduction (i.e., sexual selection and social behavior) is critical for the management of healthy, sustainable *in-situ* and *ex-situ* wildlife populations [19,20].

Musth in Asian elephants functions as a sexually selected signal to facilitate female mate choice (intersexual selection) and mediate male–male competition (intrasexual selection) [16]. Recent evidence suggests that musth serves as a roving strategy by which males traverse large areas in search of estrous females, broadcasting their sexual status to potential mates [21,22]. Female elephants appear to be more receptive to musth males [1,21]; in turn, these males should secure more mating opportunities, thus increasing their fitness over that of non-musth males. Other factors not necessarily related to musth status may also impact female preference, including age [17]. Indeed, female elephants appear to prefer to associate with older males [1,21], who are also larger because male elephants continue to grow throughout their lives [23,24]. By better characterizing the changes in the social behavior of male Asian elephants, we can more confidently identify factors like age and musth status that contribute to intersexual selection and female mate choice. Still, in polygynous species like Asian elephants, a male’s mating success can also be influenced by factors other than female choice, such as male dominance and competitive ability relative to other male conspecifics. Although younger male elephants are virtually always subordinate to older males [2], musth status seems to outweigh age-based dominance hierarchies [25,26,27], diffusing combative and potentially costly interactions between males. However, male elephants are regularly observed in groups—though in less permanent and/or defined associations than female elephants—that presumably confer some of the benefits of group-living, especially in changing environments (e.g., human-dominated landscapes) [6,8,28,29]. While few studies have investigated the factors that drive male–male sociality in elephants, there is evidence that both musth status and age interact to influence male–male group formation [2,7]; specifically, older males, especially while in musth, are generally more solitary compared to younger males, and young male elephants very commonly associate with each other during non-musth periods. To better understand why male Asian elephants exhibit flexible association patterns and the adaptive value of these male–male groups, we should characterize the social behavior (e.g., agonism, prosocial behavior, dominance behavior) exhibited by these males. Such studies would bring context to the ecological and evolutionary motivations of male elephants and why they form groups in some situations but not others. This fundamental information is required for integrative, sustainable elephant population management strategies.

Male elephants are most frequently implicated in human-elephant conflict (HEC) [30,31,32], potentially motivated to forage upon human crops as they travel longer distances searching for receptive mates. This may be especially true during musth, when males may be apt to engage in HEC to meet their energetic needs, particularly if it provides access to nutritious crops. Targeted approaches to HEC are necessary for the long-term sustainability of the endangered Asian elephant [33,34], and integrating behavioral approaches will be important in the development of conservation and mitigation plans [35]. To effectively approach HEC with a more holistic understanding of biological and ecological drivers of HEC [35,36], we suggest prioritizing efforts that seek to develop our understanding of male elephant socioecology. At the same time that *in-situ* elephant populations are threatened, *ex-situ* Asian elephant populations (i.e., those bred and housed in human care, including in zoos, private ownership, elephant camps, and other similar parks and facilities) are critical to the long-term viability of the species [37,38]; approximately one-third of the remaining global Asian elephant population exists in human care [3,39]. While *ex-situ* elephant populations have been historically female-biased, the proportion of males in captive populations is now higher than ever and will only continue to increase with enhanced breeding success [40]. In addition to better understanding male elephant socioecology to characterize the unique condition of musth, there is also a pressing need to develop best practices in housing and care for male elephants in environments that serve their physical, behavioral, and social requirements [41]. Complementary *in-situ*/*ex-situ* studies serve to inform the conservation of elephants with information from free-ranging populations, while also providing opportunities for detailed study in more controlled, managed environments. Such endeavors begin with a foundational knowledge of the natural history and social organization of elephants.

The purpose of this study was to use a complementary *in-situ*–*ex-situ* approach to identify factors that influence the social behavior of male Asian elephants, including aggression, prosocial behavior, dominance behavior, submissive behavior, and group formation. Building upon the work of long-term data on Asian elephant social organization and reproductive strategies (e.g., [1,7,21,22,42]), we aimed to contribute to growing evidence of the social complexity of male elephants using behavioral data we collected from wild elephants (observed in Wasgamuwa National Park, Sri Lanka) and zoo-housed elephants (observed in facilities across North America). Here, we describe (1) the effects of age and musth status on social group formation and composition (in wild elephants only); and (2) factors such as age, musth status, group type, and number of conspecifics that may interact and influence a range of social behaviors (in both wild and zoo-housed elephants). Specifically, (1) we hypothesized that age and musth status interact to predict the likelihood of a wild male elephant being sighted in all-male and mixed-sex groups and the time spent in those groups [6,7,21]. Additionally, (2) we hypothesized that age and musth status would be the primary drivers of changes in social behavior in all-male and mixed-sex groups in both wild and zoo-housed elephants. We anticipated the rates of aggression to be highest in all-male groups across a broad age range, and prosocial behavior would be most common in mixed-sex groups when the focal animal was older and/or in musth, consistent with predictions that musth is a roving strategy [21,22]. Because of their role in establishing and maintaining dominance hierarchies among male elephants [2,43], we hypothesized that dominance and submissive behaviors would be more common in all-male groups, with older, musth males more frequently exhibiting dominance and younger males showing submission. For all analyses involving wild and zoo-housed elephants, we expected results to be similar between populations because of the apparent importance of musth and other reproductive strategies [15,41,44].

## 2. Materials and Methods

### 2.1. Study Sites and Subjects

We observed male Asian elephants in two environments: wild elephants in Wasgamuwa National Park, Sri Lanka, and zoo-housed elephants in captive facilities in the United States. In Sri Lanka, observers collected behavioral data opportunistically on 57 days from December 2018 to April 2019—between 06:00 and 18:00 each day—from a vehicle while driving on park roads and trails. When observers encountered elephants, the composition of the group (number of adult males, adult females, juveniles, and calves) was noted; a group was defined as all elephants within 100 m of each other, moving or feeding in apparent coordination. We also photographed each adult male we encountered with a DSLR camera (Nikon D60 body fitted with AF-S VR Zoom-Nikkor 70–300 mm f/4.5–5.6 G IF-ED telephoto lens, Nikon USA) for future identification. Of 382 total elephant sightings, 256 (67.0%) included adult males. Adult male elephant sightings consisted of solitary males (*n* = 133, 52.0% of male sightings), mixed-sex groups with at least one adult male and one adult female (*n* = 83, 32.4%), and all-male groups with at least two adult males and no females (*n* = 40, 15.6%). Over the course of the study in Sri Lanka, we reliably identified 71 adult male elephants. We sighted 52 of these elephants at least once in a social group during which behavioral observations were conducted, comprising 136 observation sessions (median number of observations per elephant = 2, ranging from 1 to 10, with 47 observations in all-male groups and 89 in mixed-sex groups).

At captive facilities in the US, we conducted observations on 26 male elephants between July 2018 and April 2021 [15]. We did not alter the routine husbandry conditions (e.g., diet, feeding schedule, animal training practices, housing environments) at any facility, and all zoo elephants in this study were managed using protected contact. We aimed to conduct paired observations on each elephant, one week when the male was in musth (determined by the sustained presence of temporal gland secretions and/or urine dribbling) and one week when he was out of musth. Not all males exhibited a distinct musth episode over the study period and logistics prevented sampling all the remaining males in both musth and non-musth conditions. In total, we conducted 392 observation sessions on these elephants, with 132 sessions from 15 male elephants occurring when the focal animal was housed in a social group (median number of observations per elephant = 9, ranging from 1 to 17, with 58 observations in all-male groups and 74 in mixed-sex groups).

### 2.2. Observation Protocols

All protocols were approved by George Mason University’s IACUC (1168839-1) and all other participating facilities; permission for fieldwork in Sri Lanka was obtained from the Department of Wildlife Conservation (WL/3/2/57/18). We aimed to conduct behavioral observations on wild and zoo-housed elephants in a similar fashion. At the beginning of each observation, we noted the male’s visible musth features [the extent of temporal gland secretions (TGS) and urine dribbling (UD), using published standards [45]], later classifying a male’s musth status as non-musth, pre-musth, full-musth, and post-musth based on behavioral descriptions by LaDue et al. [15]. In Asian elephants, both TGS and UD are almost always only exhibited by males in musth, and so these signs can be used to quickly ascertain musth status in the field [46]. While the age of each zoo-housed elephant was known (mean ± SD age at the beginning of the study = 25.71 ± 15.85 years, ranging from 8.28 to 56.01 years), we estimated each wild male elephant’s age using criteria from Varma et al. [47]: 10 to 15 years, 15 to 20 years, 20 to 30 years, 30 to 40 years, 40+ years.

For this study, we only included observation sessions during which the focal animal was recorded in a group with at least one other elephant, either in a mixed-sex group (with at least one female) or an all-male group (at least one additional adult male and no females). On wild elephants, we conducted observations of ~15 min (mean ± SD observation time = 12.5 ± 3.6 min), and the order of observation in multi-male groups was determined randomly. If time permitted, we performed a maximum of three observations of an elephant during each sighting; for groups with multiple males, the time spent observing each male was equal to minimize the oversampling of individual elephants. Observations on zoo-housed elephants lasted 60 min, minus any time the animal was unexpectedly under the control of a handler (mean ± SD observation time = 58.5 ± 2.9 min). At each site visit to a US elephant facility, we aimed to conduct two observations daily—once in the morning and once in the afternoon—over a five-day period on each male elephant, totaling a maximum of ten observation sessions (i.e., ten hours) per elephant per visit. For both wild and zoo-housed elephants, we conducted observations live using ZooMonitor (Tracks^®^ Software and Lincoln Park Zoo) on a touchscreen tablet [48]. After the COVID-19 pandemic began in March 2020, we used video recordings collected by zoo staff to observe zoo-housed elephants. All observations for both wild and zoo-housed elephants were conducted by the same observer (C.A.L.); intraobserver reliability at the beginning and end of the study was confirmed with >95% agreement via an index of concordance [49].

During observations of both wild and zoo-housed elephants, we utilized all-occurrence focal animal sampling [50] for social behaviors of interest (Table 1). We classified each of these social behaviors into four broader categories: aggression, prosocial behavior, dominance behavior, and submissive behavior. All social behaviors (e.g., displace, lead, push) involved a sender/initiator and receiver, and most were classified differently based on whether the focal animal was the sender or receiver of the behavior. All social behaviors—whether the focal animal was the sender or receiver—were included in our analyses.

### 2.3. Data Analysis

We used R version 4.1.0 [51] to conduct all analyses, including the following packages: *AICcmodavg* [52], *lme4* [53], *MuMIn* [54], and *tidyverse* [55].

#### 2.3.1. Effect of Age on Musth

Our dataset prevented us from carrying out regression-type analyses to investigate the effect of age on the occurrence of musth (e.g., low and uneven sample sizes, short-term data on wild elephants), so we conducted descriptive statistics to report the percentage of wild and zoo-housed male elephants in our sample population that we observed in musth and non-musth states. Due to the small sample size, we used a binary musth descriptor in this descriptive analysis, defining “non-musth” as a male being in either the non- or post-musth stages, and “musth” as being in either the early or full musth stages [15].

#### 2.3.2. Effect of Age and Musth Status on Wild Social Group Composition

We used a linear mixed model (LMM) approach to investigate the influence of age and musth status on group formation in wild elephants; we did not conduct this analysis on zoo-housed elephants because their social groups were not formed by choice (i.e., facility and husbandry limitations resulted in the zoo-housed elephant groups we sampled). For each identified male, we separately calculated the average number of female and male conspecifics with which he was sighted out of musth and in musth during the study period. These binary musth categories were used due to the low sample size in some age classes and are defined in the previous subsection. Then, we constructed two linear mixed models (one for the number of female conspecifics and another for male conspecifics) with number of conspecifics as the response variable, and the interaction between age class and musth status (musth or non-musth) and the main effect terms were included as fixed effects. We used individual males as replicates, and so focal male identity was included as a random factor in this model. Finally, we utilized the marginal coefficient of variation (*R*^2^*_c_*) to estimate the models’ predictive value.

To identify significant differences in the number of conspecifics (female or male) between categories defined by focal male age class and musth status, we conducted factorial analyses of variance (ANOVAs). These ANOVAs used age class and binary musth status (non-musth or musth) as fixed factors.

#### 2.3.3. Factors Influencing Male Social Behavior

To account for differences in observation lengths, we determined a rate for each behavior in the number of events per hour, calculated by multiplying the number of events in an observation session by 3600 and then dividing the product by the number of seconds during which the focal animal was visible. Because social interactions were relatively rare, we subsequently summed rates of behavior of the same category to use in our analyses: aggression, prosocial behavior, dominance behavior, and submissive behavior. Furthermore, to analyze changes in the relative frequencies of social behavior, we calculated the total number of social interactions exhibited by/to the focal animal for each observation; this total was used to determine the proportion of all social interactions dedicated to each type of social behavior (aggression, prosocial behavior, dominance behavior, and submissive behavior) during an observation.

Differences in the relative frequencies of the four social behavior categories were analyzed graphically and with descriptive statistics. To further identify factors that influence social behavior variation (including in nearest neighbor scores), we generated LMMs for wild and zoo-housed elephants separately using an information theory approach [56,57,58]. We constructed models separately for the rates of each of the four types of social behaviors (aggression, prosocial behavior, dominance behavior, and submissive behavior) as response variables. We included the following variables as fixed effects: musth status (non-musth, early musth, full musth, and post-musth, as determined by temporal gland secretions), age (actual age in years for zoo-housed elephants, estimated age class for wild elephants), group type (all-male or mixed sex), and the number of elephants present (including the focal animal). We included the focal animal as a random intercept in all models to account for repeated observations on the same animals. We developed a list of candidate models encompassing these factors, including potential interactions between musth status, age, and group type (Table S1). Then, we ranked these models via Akaike Information Criterion values, corrected for small sample sizes (AIC_c_) [56] via maximum likelihood estimation. After identifying each “best” model with this approach, we excluded non-significant variables (*p* > 0.05) with a modified *χ*^2^ test using a restricted maximum likelihood approach to identify the final model for each behavioral category and method of measurement (relative frequency and behavioral rate). We estimated the explanatory value of these final models with marginal coefficients of variation (*R*^2^*_c_*).

## 3. Results

Over the study period of each population (34 months for zoo-housed elephants, 5 months for wild elephants), we noted differences between age classes in the occurrence of musth (Figure S1). We identified 72 adult male elephants (≥10 years old) in Wasgamuwa National Park, Sri Lanka, over the study period. Fifty-five of these males were sighted only out of musth, 10 only in musth, and seven both in and out of musth. We never observed males of the two youngest age classes (10–15 years and 15–20 years, *n* = 32), or of the oldest age class (≥40 years, *n* = 2) in musth. For zoo-housed elephants, the youngest male we observed with visible musth features (i.e., temporal gland secretions and/or urine dribbling) was 11.7 years old. Of the 22 remaining zoo-housed elephants over this age, 19 exhibited musth during the study period. Thirteen zoo elephants were 20 years old or younger during the study, and nine of these males showed signs of musth. Six zoo-housed elephants were over 40 years old; five of these males exhibited musth.

### 3.1. Effect of Age and Musth Status on Wild Social Group Composition

In Wasgamuwa, we observed 2.7 ± 1.0 (average ± SD) adult males in all-male groups and 2.7 ± 2.2 adult males (and 7.9 ± 5.2 adult females) in mixed-sex groups. The total group size of all-male groups ranged from two to six elephants, and from two to 45 for mixed-sex groups (including juveniles and calves). Of the 48 sightings that included at least one musth male, 16 sightings (33.3%) occurred while the male was solitary, seven (14.6%) while the male(s) was/were in an all-male group, and 25 (52.1%) while the male(s) was/were in a mixed-sex group. However, we rarely sighted two musth males in the same group: three sightings of all-male groups consisting of two musth males and two sightings of two musth males in mixed-sexed groups; however, we did not observe direct social interactions between the males in these cases. We did not observe three or more musth males in the same group.

**Figure 1:**
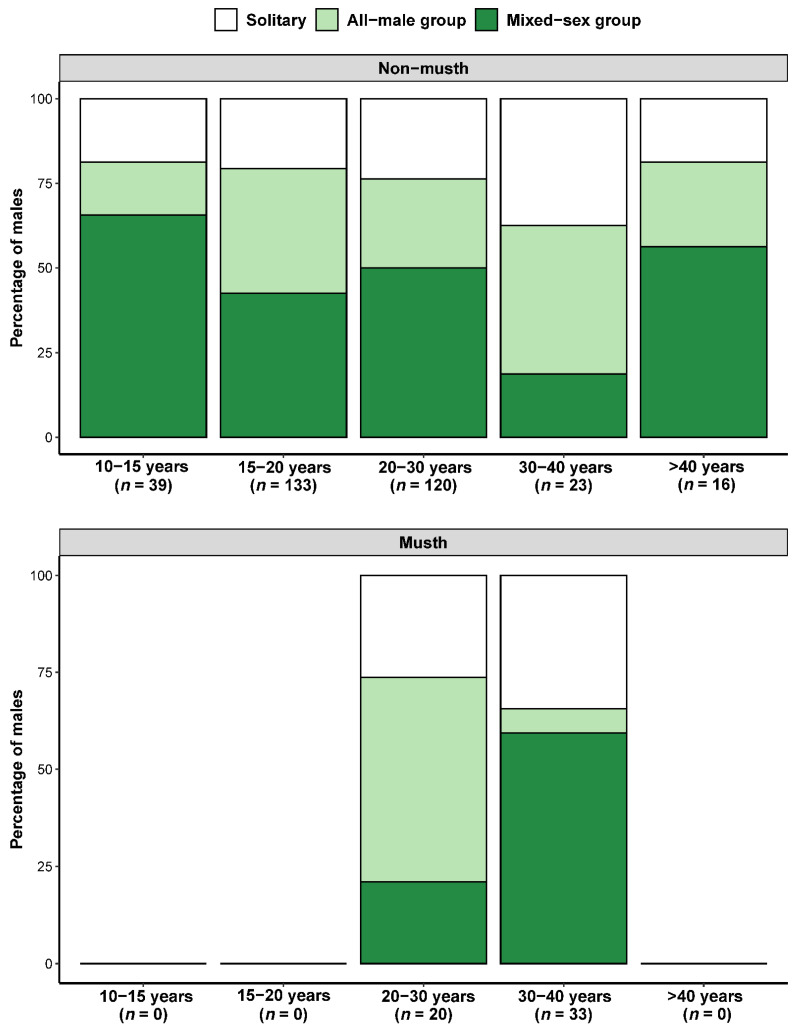
Relative frequencies of group-type sightings (solitary, all-male group, mixed-sex group) as a function of the age class and binary musth status (top panel, non-musth; bottom panel, musth) of the focal male elephants in Wasgamuwa National Park, Sri Lanka. The sample size (number of sightings) for each category is given on the horizontal axes.

Focal males that were older and in a non-musth state were generally observed more frequently solitarily or in all-male groups (and less frequently in mixed-sex groups), except for the two >40-year-old males we observed (Figure 1). For instance, non-musth males in the 10–15 years age category were recorded in mixed-sex groups in 65.6% of sightings and only 15.6% in all-male groups and 18.8% as solitary males; by 20–30 years and 30–40 years, non-musth males were only observed 50.0% or 18.8% of sightings, respectively, in mixed-sex groups, and 26.3% or 43.8% in all-male groups and 23.7% or 37.4% solitarily. However, this trend was reversed for males in musth, with a greater proportion of sightings of 30- to 40-year-old musth males in mixed-sex groups (59.4% of sightings) than 20- to 30-year-old musth males (21.1% of sightings).

**Figure 2:**
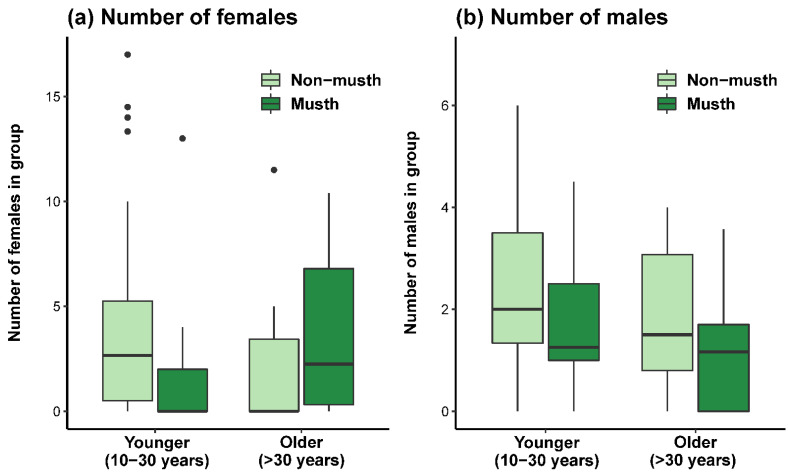
Boxplots of the association of age and binary musth status of male Asian elephants in Wasgamuwa National Park, Sri Lanka, with the number of (**a**) adult females and (**b**) adult males in a male’s group. For simplicity, ages and age classes are condensed into younger (10 to 30 years) and older (>30 years) age categories in this plot. Boxes extend from the first to the third quartile, with the median indicated by a thick line; fences extend to 1.5 times the interquartile range, and closed circles indicate values outside this range.

We found that the interaction between age and the binary musth status of a wild male elephant explained much of the variation in the number of females with which he was observed (*R*^2^*_c_* = 0.783) (Table 2a). Specifically, males in musth were sighted with more females in the group, with the number of females decreasing with the age of the male. However, a factorial ANOVA did not reveal significant differences between males due to age (*F*_4,72_ = 2.305, *p* = 0.066), musth status (*F*_1,72_ = 0.474, *p* = 0.493), or the interaction between these two factors (*F*_1,72_ = 1.523, *p* = 0.221) (Figure 2a). When we analyzed the same dataset to test the effect of age and musth status on a focal animal’s male conspecifics, our model explained less variation (*R*^2^*_c_* = 0.317) (Table 2b). Still, these factors had the opposite effect on the number of male conspecifics compared to females, with males in musth associating with fewer male conspecifics on average; the age of the focal animal had a positive or slightly negative effect on the number of male conspecifics in the group. Specifically, males in the 20–30 year age class tended to have the most male conspecifics in their social group (all other age classes were associated with fewer male conspecifics), except when the focal male was in musth; in this case, the number of male conspecifics was lower in this age class. While lack of data prevented us from further investigating the interaction between age and musth in this context, these findings were confirmed by the results of the factorial ANOVA: musth status, *F*_1,72_ = 4.482, *p* = 0.038; age, *F*_4,72_ = 0.842, *p* = 0.503; musth by age interaction, *F*_1,72_ = 0.013, *p* = 0.910 (Figure 2b).

### 3.2. Factors Influencing Male Social Behavior

**Figure 3:**
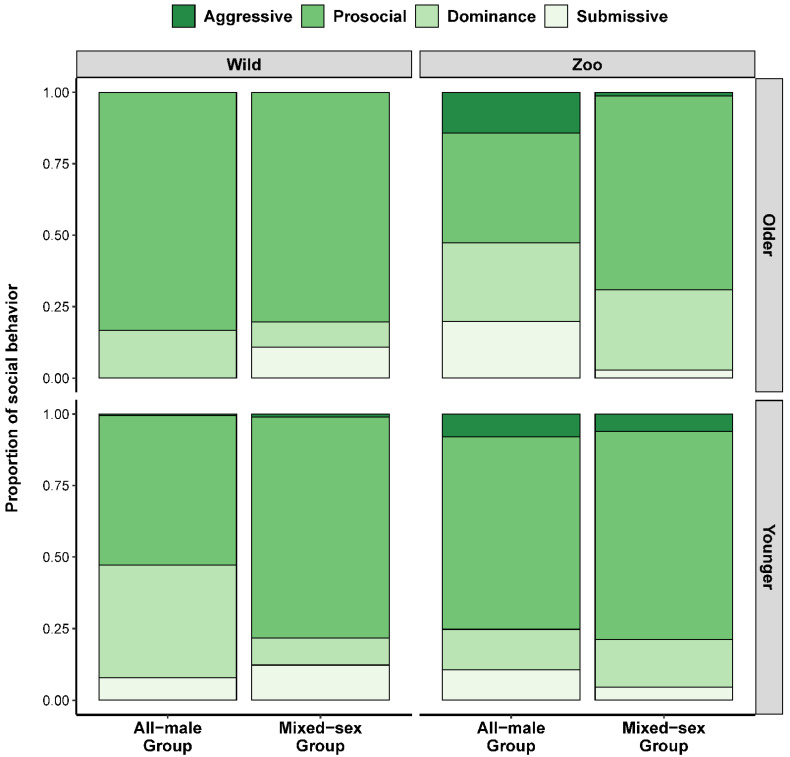
Relative frequencies of social behavior (aggression, prosocial behavior, dominance behavior, and submissive behavior) between wild and zoo-housed male Asian elephants, separated by social group-type (all-male or mixed sex) and age of focal animal (younger = 10–30 years old, older = 30+ years old). For simplicity, ages and age classes are condensed into younger and older age categories in this plot.

We found limited evidence that the relative frequencies of each type of social behavior (aggression, prosocial behavior, dominance behavior, and submissive behavior) were associated with the age and/or musth status of either wild or zoo-housed male elephants. Upon performing descriptive statistics, aggression was rare, making up on average ± SD 0.6% ± 3.2% of social behaviors exhibited by wild elephants and 6.2% ± 8.8% by zoo elephants (Figure 3). In zoo elephants, aggressive behavior was more common with the age of the focal animal and in all-male groups, but this did not appear to be true in wild elephants. The most frequently occurring social behavior in male elephants was prosocial behavior, comprising 72.5% ± 29.6% and 68.5% ± 18.9% of all social behaviors in wild and zoo-housed elephants, respectively, and it was more common in mixed-sex groups compared to all-male groups in zoo elephants (Figure 3), and in larger groups in wild elephants. Dominance and submissive behaviors were comparatively infrequent (dominance: 16.2% ± 24.2% for wild, and 18.2 ± 16.2% for zoo-housed; submissive: 10.6 ± 22.7% for wild, and 7.2% ± 10.1% for zoo-housed). Musth status was not associated with the relative frequencies of any social behavior.

**Figure 4:**
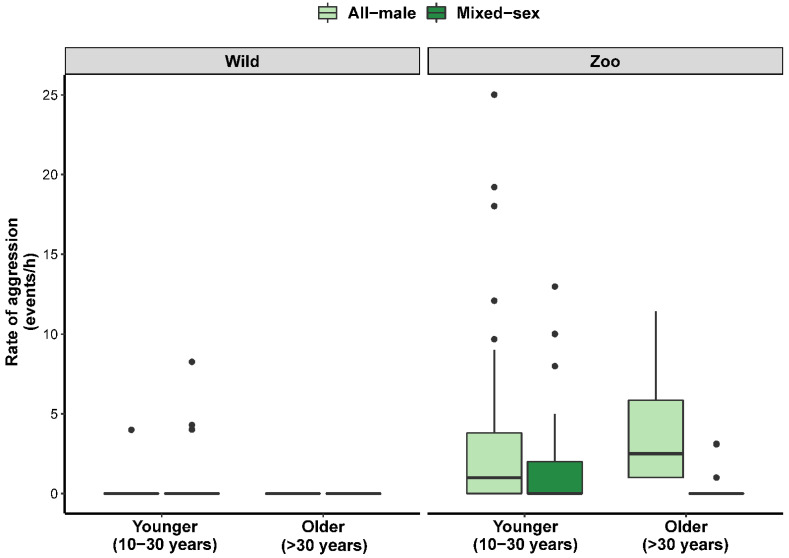
Boxplot showing the association between age and rates of aggression for wild and zoo-housed male Asian elephants in all-male and mixed-sex groups. For simplicity, ages and age classes are condensed into younger (10 to 30 years) and older (>30 years) age categories in this plot. Boxes extend from the first to the third quartile, with the median indicated by a thick line; fences extend to 1.5 times the interquartile range, and closed circles indicate values outside this range.

**Figure 5:**
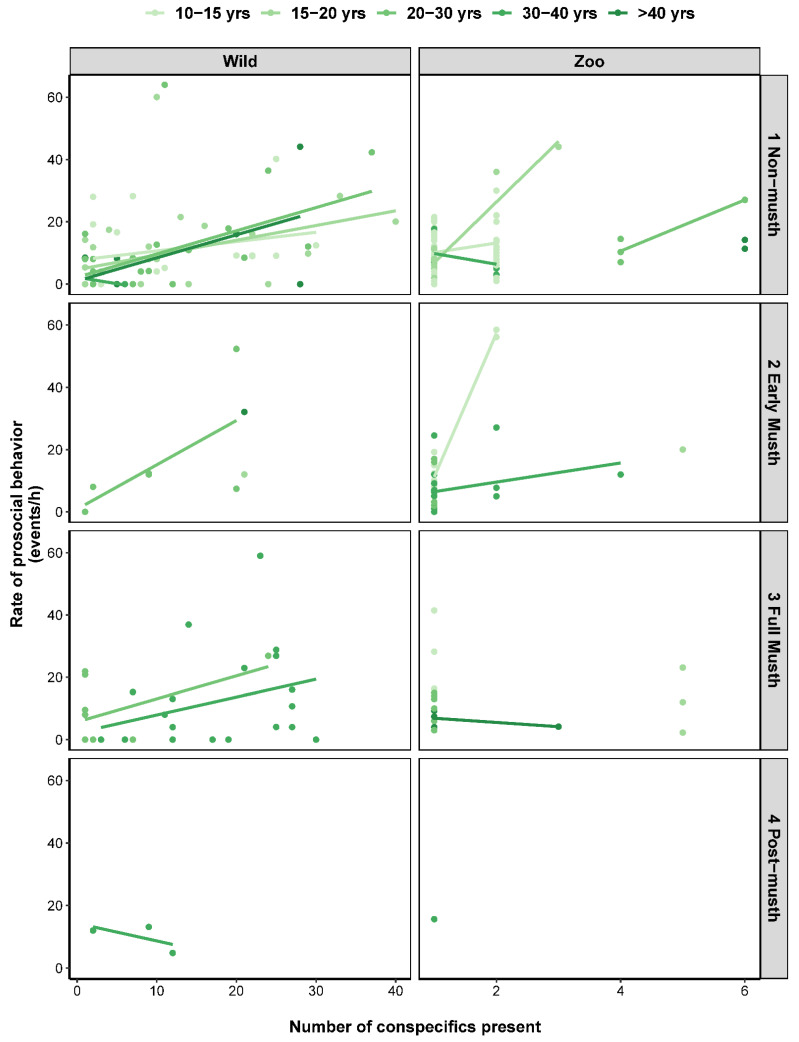
Relationship between rates of prosocial behavior and the number of conspecifics present for wild and zoo-housed male Asian elephants. Closed circles represent individual observation sessions, with regression lines shown for each age class (darker shades represent older age classes). The absence of a regression line for an age class indicates a lack of adequate data for the construction of a relationship. Note difference in scale on horizontal ax is for wild and zoo-housed elephants.

The rates of each type of social behavior reflected the relative frequencies of these behaviors described above, and when we analyzed the factors that influenced these rates, we found that various factors affected wild and zoo-housed elephants differently (Table 3, Table S2 and Table S3). Socially aggressive behaviors infrequently occurred during observations, on average ± SD for wild elephants at a rate of 0.1 ± 0.9 events/h and 2.0 ± 4.0 events/h for zoo-housed elephants. Age class predicted much of the variation in aggression rates in wild elephants (*R*^2^*_c_* = 0.371), with decreased aggression as elephants aged (Figure 4). Conversely, age was positively related to aggression rates in zoo-housed elephants—and it was less common in mixed-sex groups, especially those containing older male(s)—but the predictive value of this model was relatively weak (*R*^2^*_c_* = 0.142). Corroborating the analysis of the relative frequencies of social behavior, prosocial behaviors were much more commonly observed, with wild elephants exhibiting 9.9 ± 13.3 events/h and 11.7 ± 10.1 events/h for zoo elephants. The number of elephants present with a focal male elephant was positively related to rates of prosocial behavior in wild elephants (*R*^2^*_c_* = 0.239), while a variety of factors influenced prosocial behavior in zoo-housed elephants (*R*^2^*_c_* = 0.476): prosocial behavior increased with age and musth progression, and it was also more common in mixed-sex groups and during observations with more elephants present (Figure 5). Dominance and submissive behaviors were observed at relatively low, consistent rates across wild (dominance = 2.1 ± 4.7 events/hr; submissive = 1.3 ± 3.7 events/hr) and zoo-housed (dominance = 3.3 ± 4.7 events/hr; submissive = 2.0 ± 4.4 events/hr) elephants. Rates of submissive behavior in zoo-housed elephants were predicted by musth status (submission generally decreased in frequency as musth progressed) and were more frequent with age and in all-male groups (*R*^2^*_c_* = 0.276) (Figure S2). However, none of the factors we measured predicted submissive behavior in wild elephants. Similarly, we did not identify any contributing factors to the rates of dominance behaviors in wild or zoo-housed elephants.

## 4. Discussion

The ability of male Asian elephants to exhibit complexity in their social behavior is now well-recognized [2,6,7,8], and our study serves to characterize factors (e.g., age, musth status) that influence male elephant sociality. Further, the complementary nature of this investigation—incorporating behavioral data from both wild and zoo-housed elephants—provides context for the conservation of *in-situ* and *ex-situ* populations. Social behavior facilitates reproduction and influences movement, and so it is a research priority to understand how and why male elephants interact with conspecifics in a range of physical and social environments. To summarize our findings in this study, we observed age-based differences in the occurrence of musth between wild and zoo-housed elephant populations. Further, both age and musth status influenced group formation in wild elephants: males were less often observed in mixed-sex groups as they aged, but the reverse was true while males were in musth, indicating a strong influence of musth on the likelihood of intra- and intersexual interactions. Age and musth status were also associated with the number of male and female conspecifics in a focal male elephant’s group in the wild. When we analyzed the social behavior in *in-situ* and *ex-situ* populations, we found similar patterns. Prosocial behavior was the most observed social interaction, with aggression rarely observed in both populations. In both populations, variation in social behavior in wild elephants was associated with age and the number of conspecifics present, while even more factors were related to social behavior in zoo-housed elephants, including group type and musth status.

Musth was more commonly observed in older male elephants in the wild and in zoos. Similar to other field studies [1,21], we found that only males 20 years of age and older in Wasgamuwa National Park, Sri Lanka, entered musth, and musth was more commonly observed in the 30–40-year age class compared to the 20–30-year age class, possibly indicating musth is delayed in males until they are able to outcompete rival males for access to females [59]. Interestingly though, we observed musth in all age classes that we sampled in zoo-housed male elephants, with the youngest male to exhibit musth-like signs and behavior being approximately 11 years old [15]. It is possible that we might have observed more similar age-dependent patterns of musth at our study site in Sri Lanka with more consistent, longitudinal monitoring. These patterns are consistent with the findings of other captive studies [60,61,62]. For instance, zoo elephants that typically receive calorically rich diets may consequently be able to enter the more energetically and/or metabolically taxing state of musth at an earlier age [63,64]. Additionally, the artificial nature of zoo environments may release younger male elephants from other social or environmental factors (e.g., absence of older males, artificial social groupings) that would otherwise suppress musth until an older age [65]. More comprehensive, longitudinal field studies of Asian elephant social and reproductive behavior and physiology will lend insight into the relative contributions of each of these explanations to the patterns we observe. In doing so, *in-situ* wildlife managers can better predict how individual elephants may be motivated to behave in human-dominated landscapes, and *ex-situ* managers can optimize the care of male elephants.

We also found distinct influences of age and musth on social group formation in wild Asian elephants in Wasgamuwa. While all-male groups were typically smaller than mixed-sex groups (on average 2.7 adults versus 10.6 adults, respectively), the average number of males in either type of group (2.7 males) was similar, possibly indicating a social or ecological maximum to the number of males (but not necessarily females, which are generally smaller in body size) a group can support [66]. Furthermore, non-musth males were generally sighted less frequently in mixed-sex groups with age—and more frequently solitarily or in all-male groups—except for the oldest age class (>40 years, two elephants over 16 sightings), which was most frequently sighted in mixed-sex groups. This pattern follows what we expected: younger males disperse from their natal group between 10 and 15 years of age [67,68], and they form bachelor groups as they progress through young adulthood [7]. Even older males c. 30 years old are regularly sighted with male conspecifics, possibly serving a leadership role over younger males as has been observed in African savanna elephants [69,70,71]. However, as we hypothesized, this pattern was reversed with musth; during musth, we observed males in mixed-sex groups twice as frequently when they were 30–40 years compared to 20–30 years. These results support the clear reproductive advantage that musth confers to older males trying to locate or seeking access to mixed-sex groups, as has been reported in long-term studies of *L. africana* [43,72].

Males should preferentially associate with larger mixed-sex groups to increase the likelihood of finding a receptive female, and in turn, there should be more intense competition between males for access to larger groups [21]. We found that the interaction of age and musth status explained most of the variation in the number of females in a male’s group in Wasgamuwa, pointing to the adaptive value of musth and age in accessing receptive females. Still, the large proportion of sightings of younger musth males in all-male groups (~60% of sightings in the 20–30-year age class) suggests that musth likely also functions to influence intrasexual competition in this species [16]. In support of this prediction, we also found that the interaction of musth status and age significantly influenced the number of other adult males in a focal male’s group. Field studies of other Asian elephant populations have emphasized the competitive advantage that older, musth males have over younger males in potentially combative encounters [2,7,21]. To further qualify the adaptive consequences of this advantage, future studies should investigate the actual mating success and fitness outcomes of musth and age in *E. maximus*, and this information will be critical for conservation planning in this species.

Short-term studies like this cannot quantify such fitness outcomes in elephants, but behavioral observations can further contextualize inter- and intrasexual interactions that occur within mixed- and single-sex groups. Prosocial behavior comprised the majority of social behavior in wild and zoo-housed elephants, underscoring the dynamic social lives experienced by male Asian elephants [6,73]. Unsurprisingly, in both wild and zoo-housed elephants, we found that rates of prosocial behavior increased with the number of conspecifics present, as a higher number of potential social partners also increases the potential for social interactions to occur. Indeed, the drive to socialize with conspecifics may be a key characteristic of musth that helps explain its adaptive significance [16]. In zoo elephants, we identified a number of other factors that also affected rates of prosocial behavior, including musth status (prosocial behavior generally increased with the progression of musth), age of the focal animal (with more frequent prosocial interactions with age), and group type (more frequent prosocial behavior in mixed sex rather than all-male groups). Additionally, several of these factors (musth status, age, and group type) interacted to further explain variation in prosocial behavior in zoo-housed male elephants. These patterns support the hypothesis that musth is a roving strategy in Asian elephants [21,22], as they promote social interactions between musth males and receptive females (and the females’ groupmates). While the complex, long-lasting social bonds that female elephants are capable of forming have long received attention [4,42,73,74], these results provide further motivation to investigate the social behavior of male elephants, which may be similarly intricate and function in comparable ways. Further, while musth has customarily been associated with aggressive behavior [10,75,76,77,78], we suggest that, at least in Asian elephants, other behavioral signatures, including prosocial behavior, are much more appropriate [15]. As such, these results also emphasize the potential benefits of managing zoo-housed elephants in situations in which they have more species-appropriate opportunities for prosocial interactions [41,44,79].

In stark contrast to their reputation as highly aggressive animals [78], we infrequently observed aggression directed towards conspecifics in either wild or zoo-housed elephants, even during musth when elephants can be aggressive towards people [10,75,76,77]. The low rates of aggression we observed may be a consequence of musth’s evolved function; musth presumably serves to allow males entry into female groups (and aggression would be counterproductive to that purpose) [1,21] and to diffuse otherwise costly intrasexual interactions [2]. Nevertheless, the rate of aggressive behavior was influenced by age in both populations, but with opposite effects in each: the frequency of aggressive behavior decreased with age in wild elephants and increased with age in zoo-housed elephants (in zoo elephants, aggression was also more common in all-male groups compared to mixed-sex groups, as we predicted). Because aggression was exceedingly rare among wild elephants (aggressive events occurred only 0.09 times/h on average), we suggest caution in interpreting our results as further investigation is needed to elucidate functional patterns, but we may hypothesize that aggressive interactions are used to establish dominance hierarchies in younger male elephants but not necessarily older elephants [80]. However, in zoo-housed elephants, it is interesting that we observed higher rates of aggression in older elephants; these elephants were observed in mixed-sex and all-male groups, and yet rates of aggression remained relatively high. Historically in North America, male elephants have been housed singly, with rare access to conspecifics to allow for socialization [40]. This may be true of several of the older zoo elephants in our study, and it would further explain some of the differences between wild and zoo-housed elephant social behavior. Therefore, socialization may be an important factor in the behavioral health and development of male elephants, especially at younger ages. As more male zoo elephants are housed together [41], it will become important to monitor rates of intrasexual aggression. Based on our data, there are apparent behavioral benefits to housing males socially, and so rather than advising against providing these older males exposure to conspecifics, we suggest that zoo staff carefully monitor older male elephants when they are given social access especially those with limited social experience.

Dominance and submissive behaviors were relatively uncommon during our observations, possibly due to various communicative signals that occur between elephants that limit the need for physical interactions [16,81]. It was interesting to note that dominance behaviors were most common in wild elephants among males that were younger and in all-male groups; as such, we may hypothesize that these behaviors would be especially important in these elephants that require sociobehavioral mechanisms to resolve dominance disputes [69,82]. Our models failed to identify factors in wild or zoo-housed elephants that contribute to variation in dominance behavior among males. Similarly, we did not find influential factors for submissive behavior in wild elephants, but in zoo elephants, rates of submissive behavior were explained by musth status (decreasing with musth), age (increasing with age), group type (less common in mixed-sex groups compared to all-male groups), and the number of conspecifics present (more elephants led to more frequent submissive behavior). The ability to express dominance and submissive behaviors may be especially important for younger elephants as they learn how to socialize, and if so, zoo managers should provide opportunities for their elephants to do so. However, as these behaviors were rare, our results should again be interpreted with caution. We do not suggest that males do not exhibit dominance or submissive behaviors frequently, as both male and female elephants are known to live in multi-tiered societies, but our ethogram may not have adequately captured subtle events that serve to reinforce dominance status, such as longer-distance visual, acoustic, and/or chemical signals [16,81,83,84,85].

To develop animal-centered approaches for the conservation of *E. maximus*, long-term studies of Asian elephant behavior and social patterns are needed to follow individual elephants as they age and transition between reproductively active (e.g., musth) and inactive (e.g., non-musth) states. While we have shown here that *ex-situ* populations can also be useful for studying some aspects of social behavior—the complete life histories of these animals are often known in unparalleled detail—these populations are limited in that captive animals have limited ‘choice’ in social group formation. Additionally, artificial environments can impose unnatural pressures that make comparisons to *in-situ* populations difficult [38]. Further understanding the occurrence of social behavior and all its consequences in natural populations (e.g., group formation and structure, intrasexual competition, mate choice, fitness implications) will allow us to manage zoo-housed animals more appropriately and/or in a way that more closely resembles *in-situ* populations. In doing so, *ex-situ* populations will become better models for their *in-situ* complements, and more realistic studies (and even experimental manipulations) of zoo-housed animals will be possible, with all the existing benefits of working with closely monitored animals in human care.

## 5. Conclusions

With our unique complementary study of male elephant social behavior in *in-situ* and *ex-situ* settings, we have shown that male Asian elephants exhibit flexibility in their social behavior that is dependent upon age and musth status, among other intrinsic and extrinsic factors. This work has strong implications for managing and conserving this endangered species. The drive to reproduce powerfully influences movement and habitat use in elephants [22,86], which in turn impacts the patterns and extent of HEC [87,88]. Therefore, bolstering our understanding of sociality and reproduction in male elephants will also aid wildlife managers and conservation planners as they seek to develop sustainable landscapes for the long-term coexistence of humans and elephants. Additionally, the study of the reproductive biology of zoo-housed elephants has aided the management of wild and captive elephants in range countries [12]. As there is an evolved importance of musth and other reproductive strategies in elephants that involve dynamic social exchanges, the behavior should not be underestimated in developing captive propagation strategies in elephants or other species. Beyond this, zoos and other facilities have an inherent responsibility to provide opportunities for species-appropriate activities, including socialization. As the proportion of males in *ex-situ* elephant populations increases (males comprised only 27% of the North American population of *E. maximus* in 2017, but this will eventually approach 50% [40]), zoos must be innovative in how they manage male elephants, especially around the sexually active state of musth [41,89]. This will require an adaptive, animal-centered approach, guided by the responses of individual elephants to changes in the physical, physiological, and social environment [44,90,91]. We suggest that as zoos optimize the management of male elephants, they should reference studies like these (and forthcoming research in the field) to inform the development of holistic animal wellness programs.

## Figures and Tables

**Table 1:** Ethogram of Asian elephant social behaviors used in this study. All behaviors were classified into one of four categories: aggression, prosocial behavior, dominance behavior, and submissive behavior. For most behaviors, categories were dependent on whether the behavior was sent (initiated) or received by the focal animal; these dependencies are indicated in the columns “Send” and “Receive” with checkmarks (✓).

Category	Send	Receive	Behavior	Definition
Aggression	✓		Bite	Use teeth to contact another elephant
	✓		Head-butt	Use forehead and/or base of trunk to contact another elephant
	✓		Push	Other than with the head or trunk, use a part of the body to move another elephant (includes tusk and kick)
	✓		Spar	Elephants face each other with raised chins, pulling and pushing with intertwined trunks; contact tusk(s)/tush(es) with another elephant, accompanied by a forward lunging motion; focal animal initiates interaction
	✓		Trunk swing	Throw trunk out quickly in direction of elephant within one body length away without contact
Prosocial behavior	✓	✓	Approach	Within one body length of another elephant, locomote towards elephant, without recipient moving away
	✓	✓	Rub	Use head, body, and/or leg(s) to contact another elephant for more than one sec
	✓	✓	Trunk entwine	Wrap trunks mutually (other than in sparring context)
	✓	✓	Trunk touch	Trunk tip touches or attempts to touch another elephant on one of the following: anus, body, ear, genitals, head, mouth, temporal gland, trunk, other
Dominance behavior	✓		Chase	Rapid pursuit of another elephant that is moving away from focal animal
	✓		Displace	Move to within one body length of another elephant, who apparently leaves as a result of the proximity
	✓		Hoard	Prevent another elephant from using a resource, either actively (through quick contact) or passively (body positioning) (e.g., food, water, mud wallow)
	✓		Lead	Be followed within one body length of another elephant for at least two body lengths in distance
	✓		Steal	Take resource from another elephant that is actively consuming that resource (e.g., food, water, mud wallow), preventing them from using it
		✓	Back-up	Conspecific walks backward to within one body length of focal elephant (or rump turned toward focal elephant in proximity)
		✓	Leave	Conspecific moves away from focal elephant that is within one body length
		✓	Share	While consuming a resource, another elephant allows focal animal to use same resource (e.g., food, water, mud wallow)
Submissive behavior	✓		Back-up	Walk backward to within one body length of conspecific (or turn rump towards elephant in proximity)
		✓	Bite	Another elephant uses teeth to contact focal elephant
		✓	Chase	Conspecific rapidly pursues focal elephant, and the focal elephant is moving away from the initiator
		✓	Displace	Conspecific moves to within one body length of focal elephant, who apparently leaves as a result of the proximity
		✓	Head-butt	Conspecific uses forehead and/or base of the trunk to contact focal elephant
		✓	Hoard	Conspecific prevents focal elephant from using a resource, either actively (through quick contact) or passively (body positioning) (e.g., food, water, mud wallow)
		✓	Lead	Follow within one body length of another elephant for at least two body lengths in distance
		✓	Mount	Another elephant places forelegs on back of focal elephant
		✓	Push	Other than with the head or trunk, conspecific uses a part of the body to move focal elephant (includes tusk and kick)
		✓	Spar	Elephants face each other with raised chins, pulling and pushing with intertwined trunks; contact tusk(s)/tush(es) with another elephant, accompanied by a forward lunging motion; conspecific initiates interaction
		✓	Steal	Conspecific takes a resource from focal elephant that is actively consuming that resource (e.g., food, water, mud wallow), preventing them from using it
		✓	Trunk swing	Conspecific throws trunk out quickly in the direction of focal elephant less than one body length away without contact
		✓	Trunk over back	Conspecific places at least two-thirds of trunk over the back, head, or neck of the focal elephant

**Table 2:** Summary of linear mixed model (LMM) for number of (a) females and (b) male conspecifics (not including the focal animal) present in social groups of wild male Asian elephants in Wasgamuwa National Park, Sri Lanka. Positive estimates of fixed effects (the interaction between binary musth status and age class and their main effects) indicate a positive effect of each factor on the number of females present. For musth status, “non-musth” was the reference value, and the 10–15 year age class was the reference value for age class. Rank deficiency in the fixed effect model matrix resulted in several excluded coefficients (marked by “—”). SE = standard error.

	Fixed Effect	Estimate	SE	*t*-Value
(a) Number of females in group	Intercept	5.680	1.155	4.918
	Musth	3.305	1.393	2.373
	15–20 year	–1.948	1.461	–1.334
	20–30 year	–2.953	1.426	–2.070
	30–40 year	–5.060	1.785	–2.834
	>40 year	1.785	3.055	0.584
	Musth: 15–20 year	—	—	—
	Musth: 20–30 year	–4.195	1.804	–2.325
	Musth: 30–40 year	—	—	—
	Musth: >40 year	—	—	—
(b) Number of males in group	Intercept	2.088	0.451	4.626
	Musth	–0.791	0.759	–1.042
	15–20 year	–0.173	0.571	–0.304
	20–30 year	0.632	0.565	–1.119
	30–40 year	–0.056	0.739	–0.075
	>40 year	0.198	1.194	–0.166
	Musth: 15–20 year	—	—	—
	Musth: 20–30 year	–0.433	0.965	–0.448
	Musth: 30–40 year	—	—	—
	Musth: >40 year	—	—	—

**Table 3:** Summary of linear mixed models (LMMs) identified via AIC-guided model selection procedures for rates of social behavior in wild and zoo elephants, constructed separately. Positive estimates of fixed effects indicate a positive effect of each factor on the rate of each type of social behavior (aggression, prosocial behavior, dominance behavior, and submissive behavior). Age class was used to estimate the age of wild elephants (10–15 years, the youngest age class, was used as the reference value), while the exact age of each zoo-housed elephant measured in years was known. Group type (“Group”) was either all-male (the reference value) or mixed sex. The factor “Eles present” was measured by the number of conspecifics present during the observation. Musth status was defined by non-musth (reference value), early musth (“Early”), full musth (“Full”), or post-musth. Est. = Estimate; SE = standard error.

	WILD	Est.	SE	*t*-Value	ZOO	Est.	SE	*t*-Value
Rate aggression	Intercept	0.922	0.252	3.660	Intercept	0.545	1.565	0.987
	15–20 years	−0.751	0.314	−2.393	Age	0.118	0.105	1.123
	20–30 years	−0.916	0.310	−2.954	Group (mixed)	−0.964	1.910	−0.505
	30–40 years	−0.922	0.354	−2.605	Age:Group	0.167	0.111	1.508
	40+ years	−0.922	0.532	−1.731				
Rate prosocial behavior	Intercept	3.985	1.541	2.586	Intercept	6.387	3.806	1.678
	Eles present	0.596	0.106	5.646	Early musth	−34,250	7711	−4.441
					Full musth	29,260	24,320	1.203
					Post-musth	13,230	9.044	1.463
					Age	0.0174	0.244	0.071
					Group (mixed)	4.292	4.452	0.964
					Eles present	3.968	0.806	4.921
					Early musth:Age	2920	657.5	4.442
					Full musth:Age	−2418	2010	−1.202
					Early:Group	34,250	7712	4.441
					Full:Group	−29,270	24,320	−1.204
					Age:Group	−0.406	0.257	−1.577
					Early:Group:Age	−2920	657.5	−4.442
					Full:Age:Group	2418.0	2010	1.203
Rate dominance behavior	Intercept	2.102	0.403	5.218	Intercept	3.257	0.423	7.707
Rate submissive behavior	Intercept	1.316	0.360	3.652	Intercept	2.297	1.498	1.534
					Early musth	−19,420	3853	−5.041
					Full musth	−4.456	12,210	−0.365
					Post-musth	−0.254	4.079	−0.062
					Age	0.079	0.100	0.791
					Group (mixed)	−0.206	1.973	−0.104
					Eles present	1656	328.5	5.042
					Early musth:Age	367.9	1009	0.364
					Full musth:Age	19,420	3853	5.041
					Early:Group	4453	12,210	0.365
					Full:Group	−0.138	0.116	−1.191
					Age:Group	−1656	328.5	−5.042
					Early:Group:Age	−367.9	1009	−0.364

## Data Availability

Data are available from the corresponding author upon reasonable request.

## Supplementary Materials

The following supporting information can be downloaded at: https://www.mdpi.com/article/10.3390/ani12091215/s1. The following supporting information is included as supplementary material: Table S1, List of candidate models used during linear mixed model (LMM) approach to identify factors (including potentially interacting effects) that contributed to variation in the rate of social behaviors (aggression, prosocial behavior, dominance behavior, submissive behavior). Models were constructed separately for each behavior, and for wild and zoo-housed elephants. All models included focal animal identity as a random factor; Figure S1, Percentage of male Asian elephants in Wasgamuwa National Park, Sri Lanka (top panel), and in US zoos (bottom panel) of various age classes that were sighted in and/or out of musth over the study period (wild elephants, December 2018 to April 2019; zoo elephants, July 2018 to April 2021). The number of unique males in each age class are given on the horizontal axis. Each male is represented only once; Table S2, Ranked regression models investigating effect of various factors on rates of social behavior in zoo-housed elephants. Other statistics include parameterization (*k*), log-likelihood (LL), Akaike’s Information Criterion score (AIC_c_), differences in AIC_c_ (ΔAIC_c_), Akaike weight (*wi*), and cumulative Akaike weights (cum. *wi*) calculated with restricted maximum likelihood estimation. Refer to Table S1 for descriptions of each parameter. Asterices (*) indicate term(s) from the best model that were dropped for non-significance in the “best” model; marginal coefficients of determination (*R*^2^*_c_*) are given for the best model after non-significant term(s) were dropped; Table S3, Ranked regression models investigating effect of various factors on rates of social behavior in wild elephants. Other statistics include parameterization (*k*), log-likelihood (LL), Akaike’s Information Criterion score (AIC_c_), differences in AICc (ΔAIC_c_), Akaike weight (*wi*), and cumulative Akaike weights (cum. *wi*) calculated with restricted maximum likelihood estimation. Refer to Table S1 for descriptions of each parameter. Asterices (*) indicate term(s) from the best model that were dropped for non-significance in the “best” model; marginal coefficients of determination (*R*^2^*_c_*) are given for the best model after non-significant term(s) were dropped; Figure S2, Relationship between rates of submissive behavior and the number of conspecifics present during, for wild and zoo-housed male Asian elephants. Closed circles represent individual observation sessions, with regression lines shown for each age class (darker shades represent older age classes). Absence of a regression line for an age class indicates lack of adequate data for construction of a relationship. Note difference in scale on horizontal axis for wild and zoo-housed elephants.

## References

[1] Chelliah K., Sukumar R. (2015). Interplay of male traits, male mating strategies and female mate choice in the Asian elephant. Elephas Maximus Behav..

[2] Chelliah K., Sukumar R. (2013). The role of tusks, musth and body size in male-male competition among Asian elephants. Elephas Maximus Anim. Behav..

[3] Sukumar R. (2003). The Living Elephants: Evolutionary Ecology, Behavior, and Conservation.

[4] de Silva S., Ranjeewa A.D.G., Kryazhimskiy S. (2011). The dynamics of social networks among female Asian elephants. BMC Ecol..

[5] Nandini S., Keerthipriya P., Vidya T.N.C. (2018). Group size differences may mask similarities in social structure: A comparison of female elephant societies. Behav. Ecol..

[6] Srinivasaiah N., Kumar V., Vaidyanathan S., Sukumar R. (2019). All-male groups in Asian elephants: A novel, adaptive social strategy in increasingly anthropogenic landscapes of southern India. Sci. Rep..

[7] Keerthipriya P., Nandini S., Vidya T.N.C. (2021). Effects of male age and female presence on male associations in a large, polygynous mammal in southern India: The Asian elephant. Front. Ecol. Evol..

[8] Katugaha H.I.E., Silva M.D., Santiapillai C. (1999). A long-term study on the dynamics of the elephant (*Elephas maximus*) population in Ruhuna National Park, Sri Lanka. Biol. Conserv..

[9] Eisenberg J.F., McKay G.M., Jainudeen M.R. (1971). Reproductive behavior of the Asiatic elephant (*Elephas maximus maximus* L.). Behaviour.

[10] Jainudeen M.R., McKay G.M., Eisenberg J.F. (1972). Observations on musth in the domesticated Asiatic elephant (*Elephas maximus*). Mammalia.

[11] Jainudeen M.R., Katongole C.B., Short R.V. (1972). Plasma testosterone levels in relation to musth and sexual activity in the male Asiatic elephant. Elephas Maximus J. Reprod. Fertil..

[12] Brown J.L., Holt W.V., Brown J.L., Comizzoli P. (2014). Comparative reproductive biology of elephants. Reproductive Sciences in Animal Conservation: Progress and Prospects.

[13] Brown J.L., Somerville M., Riddle H.S., Keele M., Duer C.K., Freeman E.W. (2007). Comparative endocrinology of testicular, adrenal and thyroid function in captive Asian and African elephant bulls. Gen. Comp. Endocrinol..

[14] Chave E., Edwards K.L., Paris S., Prado N., Morfeld K.A., Brown J.L. (2019). Variation in metabolic factors and gonadal, pituitary, thyroid, and adrenal hormones in association with musth in African and Asian elephant bulls. Gen. Comp. Endocrinol..

[15] LaDue C.A., Vandercone R.P.G., Kiso W.K., Freeman E.W. (2022). Behavioral characterization of musth in Asian elephants (*Elephas maximus*): Defining progressive stages of male sexual behavior in *in-situ* and *ex-situ* populations. Appl. Anim. Behav. Sci..

[16] LaDue C.A., Schulte B.A., Kiso W.K., Freeman E.W. (2022). Musth and sexual selection in elephants: A review of signaling properties and potential fitness consequences. Behaviour.

[17] Hollister-Smith J.A., Poole J.H., Archie E.A., Vance E.A., Georgiadis N.J., Moss C.J., Alberts S.C. (2007). Age, musth and paternity success in wild male African elephants, *Loxodonta Africana*. Anim. Behav..

[18] Rasmussen H.B., Okello J.B.A., Wittemyer G., Siegismund H.R., Arctander P., Vollrath F., Douglas-Hamilton I. (2008). Age- and tactic-related paternity success in male African elephants. Behav. Ecol..

[19] Swaisgood R.R., Schulte B.A., Kleiman D.G., Thompson K.V. (2010). Applying knowledge of mammalian social organization, mating systems, and communication to management. Wild Mammals in Captivity: Principles and Techniques for Zoo Management.

[20] Schulte-Hostedde A.I., Mastromonaco G.F. (2015). Integrating evolution in the management of captive zoo populations. Evol. Appl..

[21] Keerthipriya P., Nandini S., Gautam H., Revathe T., Vidya T.N.C. (2020). Musth and its effects on male–male and male–female associations in Asian elephants. J. Mammal..

[22] Fernando P., Wikramanayake E.D., Janaka H.K., Jayasinghe L.K.A., Gunawardena M., Kotagama S.W., Weerakoon D., Pastorini J. (2008). Ranging behavior of the Asian elephant in Sri Lanka. Mamm. Biol..

[23] Sukumar R., Joshi N.V., Krishnamurthy V. (1988). Growth in the Asian elephant. Proc. Indian Acad. Sci. (Anim. Sci.).

[24] Mumby H.S., Chapman S.N., Crawley J.A.H., Mar K.U., Htut W., Soe A.T., Aung H.H., Lummaa V. (2015). Distinguishing between determinate and indeterminate growth in a long-lived mammal. BMC Evol. Biol..

[25] Rasmussen L.E.L., Krishnamurthy V. (2000). How chemical signals integrate Asian elephant society: The known and the unknown. Zoo Biol..

[26] Rasmussen L.E.L., Riddle H.S., Krishnamurthy V. (2002). Mellifluous matures to malodorous in musth. Nature.

[27] Greenwood D.R., Comeskey D., Hunt M.B., Rasmussen L.E.L. (2005). Chirality in elephant pheromones. Nature.

[28] Chiyo P.I., Moss C.J., Alberts S.C. (2012). The influence of life history milestones and association networks on crop-raiding behavior in male African elephants. PLoS ONE.

[29] Chiyo P.I., Wilson J.W., Archie E.A., Lee P.C., Moss C.J., Alberts S.C. (2014). The influence of forage, protected areas, and mating prospects on grouping patterns of male elephants. Behav. Ecol..

[30] Ekanayaka S.K.K., Campos-Arceiz A., Rupasinghe M., Pastorini J., Fernando P. (2011). Patterns of crop raiding by Asian elephants in a human-dominated landscape in southeastern Sri Lanka. Gajah.

[31] Sukumar R., Gadgil M. (1988). Male-female differences in foraging on crops by Asian elephants. Anim. Behav..

[32] LaDue C.A., Eranda I., Jayasinghe C., Vandercone R.P.G. (2021). Mortality patterns of Asian elephants in a region of human–Elephant conflict. J. Wildl. Manag..

[33] Williams C., Tiwari S.K., Goswami V.R., de Silva S., Kumar A., Baskaran N., Yoganand K., Menon V. (2020). Elephas maximus. The IUCN Red List of Threatened Species.

[34] Menon V., Tiwari S.K. (2019). Population status of Asian elephants *Elephas maximus* and key threats. Int. Zoo Yearb..

[35] Mumby H.S., Plotnik J.M. (2018). Taking the elephants’ perspective: Remembering elephant behavior, cognition and ecology in human-elephant conflict mitigation. Front. Ecol. Evol..

[36] AsERSM (2017). Asian Elephant Range States Meeting Final Report.

[37] Conley S. (2019). Conservation philosophy and activities of the International Elephant Foundation. Int. Zoo Yearb..

[38] Hutchins M. (2006). Variation in nature: Its implications for zoo elephant management. Zoo Biol..

[39] Sukumar R. (2006). A brief review of the status, distribution and biology of wild Asian elephants. Int. Zoo Yearb..

[40] Nordin C. (2017). Asian Elephant-2016 North American Regional Studbook.

[41] Hartley M., Wood A., Yon L. (2019). Facilitating the social behaviour of bull elephants in zoos. Int. Zoo Yearb..

[42] Vidya T.N.C., Sukumar R. (2005). Social organization of the Asian elephant (*Elephas maximus*) in southern India inferred from microsatellite DNA. J. Ethol..

[43] Poole J.H. (1987). Rutting behavior in African elephants: The phenomenon of musth. Behaviour.

[44] Schreier A.L., Readyhough T.S., Moresco A., Davis M., Joseph S. (2021). Social dynamics of a newly integrated bachelor group of Asian elephants (*Elephas maximus*): Welfare implications. J. Appl. Anim. Welf. Sci..

[45] Finnell S., Glaeser S. (2016). Asian Elephant Musth Scale-Visible Signs.

[46] Rasmussen L.E.L., Krishnamurthy V. (2001). Urinary, temporal gland, and breath odors from Asian elephants of Mudumalai National Park. Gajah.

[47] Varma S., Baskaran N., Sukumar R. (2012). Field Key for Elephant Population Estimation and Age and Sex Classification: Resource Material for Synchronized Elephant Population Count Using Block Count, Line Transect Dung Count Method and Waterhole Count.

[48] Wark J.D., Cronin K.A., Niemann T., Shender M.A., Horrigan A., Kao A., Ross M.R. (2019). Monitoring the behavior and habitat use of animals to enhance welfare using the ZooMonitor app. Anim. Behav. Cogn..

[49] Bateson M., Martin P. (2021). Measuring Behaviour: An Introductory Guide.

[50] Altmann J. (1974). Observational study of behavior: Sampling methods. Behaviour.

[51] R Core Team (2021). R: A Language and Environment for Statistical Computing.

[52] Mazerolle M.J. AICcmodavg: Model Selection and Multimodel Inference Based on (Q)AIC(c); R package version 2.2-2; 2019. https://cran.r-project.org/web/packages/AICcmodavg/index.html.

[53] Bates D., Maechler M., Bolker B., Walker S. (2015). Fitting linear mixed-effects models using lme4. J. Stat. Softw..

[54] Bartón K. MuMIn: Multi-Model Inference; 1.43.15; 2019. https://cran.r-project.org/web/packages/lme4/index.html.

[55] Wickham H., Averick M., Bryan J., Chang W., McGowan L.D.A., François R., Grolemund G., Hayes A., Henry L., Hester J. (2019). Welcome to the tidyverse. J. Open Source Softw..

[56] Burnham K.P., Anderson D.R. (2002). Model Selection and Multimodel Inference: A Practical Information-Theoretic Approach.

[57] Johnson J.B., Omland K.S. (2004). Model selection in ecology and evolution. Trends Ecol. Evol..

[58] Zuur A.F., Ieno E.N. (2016). A protocol for conducting and presenting results of regression-type analyses. Methods Ecol. Evol..

[59] Whitehead H. (1994). Delayed competitive breeding in roving males. J. Theor. Biol..

[60] LaDue C.A., Scott N.L., Margulis S.W. (2014). A survey of musth among captive male elephants in North America: Updated results and implications for management. J. Elephant Manag. Assoc..

[61] Scott N.L., Riddle H. (2003). Assessment of musth in captivity: A survey of factors affecting the frequency and duration of musth in captive male elephants *Elephas Maximus-Loxodonta Africana*. J. Elephant Manag. Assoc..

[62] Kurt F., Garaï M.E. (2007). The Asian Elephant in Captivity: A Field Study.

[63] Silva I.D., Kuruwita V.Y. (1993). Hematology, plasma, and serum biochemistry values in free-ranging elephants (*Elephas maximus ceylonicus*) in Sri Lanka. J. Zoo Wildl. Med..

[64] Dickerman R.D., Pernikoff D., Zachariah N.Y., McConathy W.J., Gracy R.W., Raven P.V. (1994). Creatinine kinase and lactic dehydrogenase isozyme measurements in male Asian elephants (*Elephas maximus*) during musth and nonmusth. Clin. Chem..

[65] Slotow R., van Dyk G., Poole J., Page B., Klocke A. (2000). Older bull elephants control young males. Nature.

[66] Ruckstuhl K.E., Neuhaus P. (2002). Sexual segregation in ungulates: A comparative test of three hypotheses. Biol. Rev..

[67] Arivazhagan C., Sukumar R. (2008). Constructing age structures of Asian elephant populations: A comparison of two field methods of age estimation. Gajah.

[68] Sukumar R. (1989). Ecology of the Asian elephant in southern Indian. I. Movement and habitat utilization patterns. J. Trop. Ecol..

[69] Evans K.E., Harris S. (2008). Adolescence in male African elephants, *Loxodonta africana*, and the importance of sociality. Anim. Behav..

[70] Goldenberg S.Z., de Silva S., Rasmussen H.B., Douglas-Hamilton I., Wittemyer G. (2014). Controlling for behavioural state reveals social dynamics among male African elephants, *Loxodonta africana*. Anim. Behav..

[71] Allen C.R.B., Brent L.J.N., Motsentwa T., Weiss M.N., Croft D.P. (2020). Importance of old bulls: Leaders and followers in collective movements of all-male groups in African savannah elephants (*Loxodonta africana*). Sci. Rep..

[72] Poole J.H., Lee P.C., Njiraini N., Moss C.J., Moss C.J., Croze H., Lee P.C. (2011). Longevity, competition, and musth: A long-term perspective on male reproductive strategies. The Amboseli Elephants: A Long-Term Perspective on a Long-Lived Mammal.

[73] Sukumar R. (1989). The Asian Elephant: Ecology and Management.

[74] Nandini S., Keerthipriya P., Vidya T.N.C. (2017). Seasonal variation in female Asian elephant social structure in Nagarahole-Bandipur, southern India. Anim. Behav..

[75] Brown J.L., Corea R., Dangolla A., Easwaran E.K., Mikota S., Oo Z.M., Sarma K., Thitaram C. (2020). Management and care of captive Asian elephant bulls in musth. Gajah.

[76] Rajaram A. (2006). Musth in elephants. Resonance.

[77] Santiapillai C., Read B., Jacobson G., Wijeyamohan S., Rambukpotha S. (2011). A paradigm shift in the management of musth among bull elephants in captivity in Sri Lanka. Ceylon J. Sci. (Biol. Sci.).

[78] Gore M., Hutchins M., Ray J. (2006). A review of injuries caused by elephants in captivity: An examination of predominant factors. Int. Zoo Yearb..

[79] Readyhough T.S., Joseph S., Davis M., Moresco A., Schreier A.L. (2022). Impacts of socialization on bull Asian elephant (*Elephas maximus*) stereotypical behavior. J. Zool. Bot. Gard..

[80] Keerthipriya P. (2018). Associations, Dominance Interactions, and Musth in Male ASIAN Elephants in Nagarahole and Bandipur National Parks, Southern India.

[81] Schulte B.A., Rasmussen L.E.L. (1999). Signal-receiver interplay in the communication of male condition by Asian elephants. Anim. Behav..

[82] Evans K., Moore R., Harris S. (2013). The social and ecological integration of captive-raised adolescent male African elephants (*Loxodonta africana*) into a wild population. PLoS ONE.

[83] Schulte B.A., LaDue C.A. (2021). The chemical ecology of elephants: 21st century additions to our understanding and future outlooks. Animals.

[84] Stoeger A.S., Rosenfeld C.S., Hoffmann F. (2021). Elephant sonic and infrasonic sound production, perception, and processing. Neuroendocrine Regulation of Animal Vocalization: Mechanisms and Anthropogenic Factors in Animal Communication.

[85] de Silva S. (2010). Acoustic communication in the Asian elephant. Elephas Maximus Maximus Behav..

[86] Taylor L.A., Vollrath F., Lambert B., Lunn D., Douglas-Hamilton I., Wittemyer G. (2020). Movement reveals reproductive tactics in male elephants. J. Anim. Ecol..

[87] Fernando P., De Silva M.K.C.R., Jayasinghe L.K.A., Janaka H.K., Pastorini J. (2021). First country-wide survey of the endangered Asian elephant: Towards better conservation and management in Sri Lanka. Oryx.

[88] Sukumar R. (1991). The management of large mammals in relation to male strategies and conflict with people. Biol. Conserv..

[89] Schulte B.A. (2000). Social structure and helping behavior in captive elephants. Zoo Biol..

[90] Scott N.L., LaDue C.A. (2019). The behavioral effects of exhibit size versus complexity in African elephants: A potential solution for smaller spaces. Zoo Biol..

[91] Thevarajah S.J., Readyhough T.S., Davis M., Moresco A., Joseph S., Schreier A.L. (2021). Nighttime behavior and the length of social relationships in male Asian elephants. J. Appl. Anim. Welf. Sci..

