# Supplementary material for: Social Behavior and Group Formation in Male Asian Elephants (Elephas maximus): The Effects of Age and Musth in Wild and Zoo-Housed Animals
Source: Animals (Basel). 2022 May 8;12(9):1215. doi: 10.3390/ani12091215 (PMC9100748; doi:10.3390/ani12091215)
Supplement: Supplementary file 1 [file animals-12-01215-s001.zip › animals-1706702-supplementary.pdf]

## Supplementary Materials

**Social behavior and group formation in male Asian elephants (*Elephas maximus*): The effects of age and musth in wild and zoo-housed animals**

Chase A. LaDue, Rajnish P.G. Vandercone, Wendy K. Kiso, and Elizabeth W. Freeman

**Table S1.** List of candidate models used during linear mixed model (LMM) approach to identify factors (including potentially interacting effects) that contributed to variation in the rate of social behaviors (aggression, prosocial behavior, dominance behavior, submissive behavior). Models were constructed separately for each behavior, and for wild and zoo-housed elephants. All models included focal animal identity as a random factor.

| No. | Model structure |
|---|---|
| 1 | Behavior rate ~ Null |
| 2 | Behavior rate ~ Musth status † |
| 3 | Behavior rate ~ Musth status + Age ‡ |
| 4 | Behavior rate ~ Musth status × Age |
| 5 | Behavior rate ~ Musth status + Age + Group type § |
| 6 | Behavior rate ~ Musth status × Age × Group type |
| 7 | Behavior rate ~ Musth status + Age + Group type + Elephants present ¶ |
| 8 | Behavior rate ~ Musth status × Age × Group type + Elephants present |
| 9 | Behavior rate ~ Age |
| 10 | Behavior rate ~ Age + Group type |
| 11 | Behavior rate ~ Age × Group type |
| 12 | Behavior rate ~ Age + Group type + Elephants present |
| 13 | Behavior rate ~ Age × Group type + Elephants present |

† Categorical variable: Non-musth (reference value), Early musth, Full musth, or Post-musth

‡ Age class for wild elephants, and age in years for zoo-housed elephants

§ Categorical variable: All-male group (reference value) or Mixed-sex group

¶ Number of elephants present in same group during observation

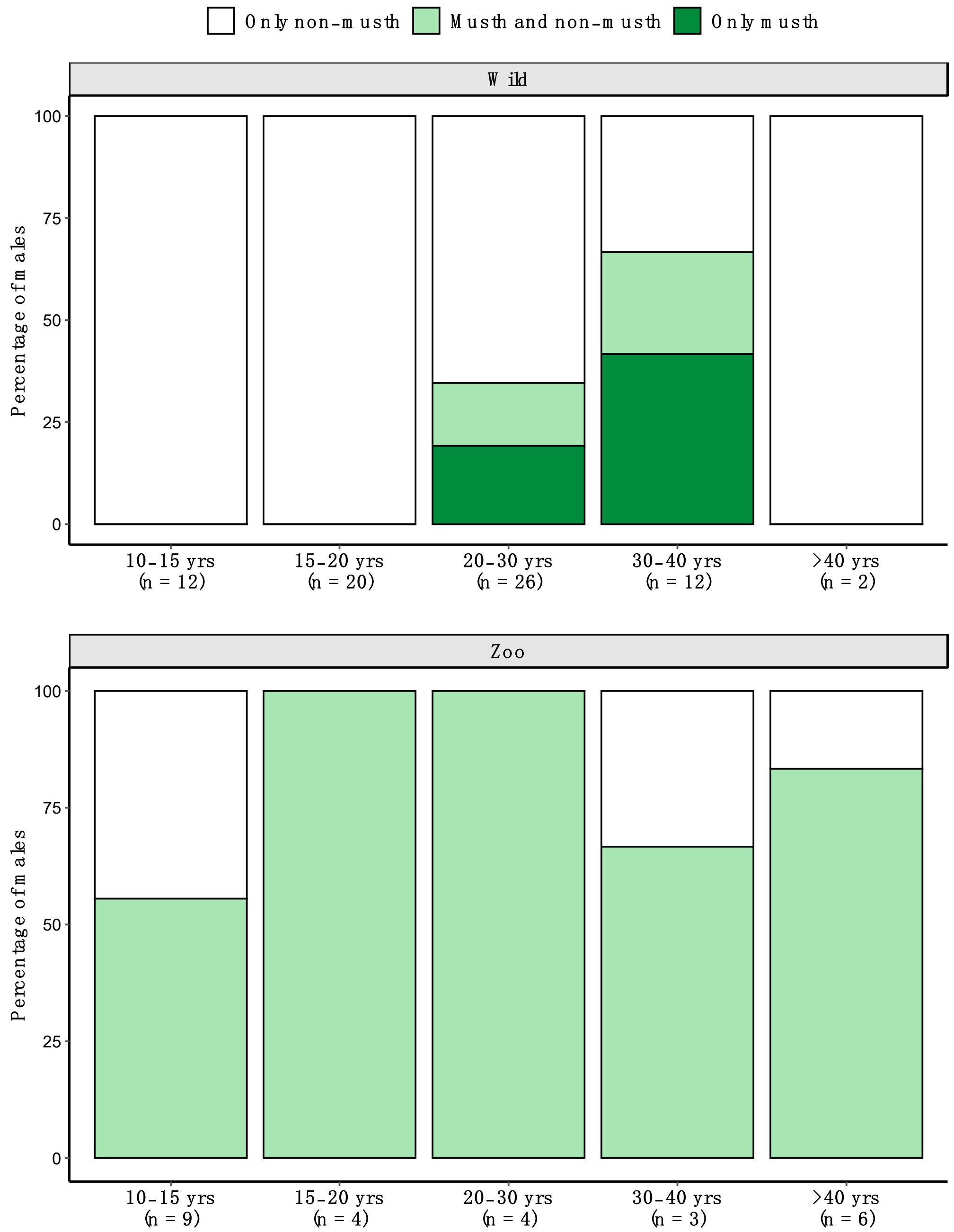


**Figure S1.** Percentage of male Asian elephants in Wasgamuwa National Park, Sri Lanka (top panel), and in US zoos (bottom panel) of various age classes that were sighted in and/or out of musth over the study period (wild elephants, December 2018 to April 2019; zoo elephants, July 2018 to April 2021). The number of unique males in each age class are given on the horizontal axis. Each male is represented only once.

**Table S2.** Ranked regression models investigating effect of various factors on rates of social behavior in zoo-housed elephants. Other statistics include parameterization ($k$), log-likelihood (LL), Akaike's Information Criterion score ($AIC_c$), differences in $AIC_c$ ($\Delta AIC_c$), Akaike weight ($w_i$), and cumulative Akaike weights (cum. $w_i$) calculated with restricted maximum likelihood estimation. Refer to Table S1 for descriptions of each parameter. Asterices (*) indicate term(s) from the best model that were dropped for non-significance in the "best" model; marginal coefficients of determination ($R^2_c$) are given for the best model after non-significant term(s) were dropped.

| **Zoo elephants: Rates of social behavior** | ***k*** | **$AIC_c$** | **$\Delta AIC_c$** | **$w_i$** | **LL** | **cum. $w_i$** |
|---|---|---|---|---|---|---|
| **Aggression: $R^2_c$ = 0.142** | | | | | | |
| Age × Group type | 6 | 741.19 | 0.00 | 0.17 | −364.26 | 0.17 |
| Age + Group type | 5 | 741.40 | 0.20 | 0.15 | −365.46 | 0.32 |
| Age × Group type + Elephants present | 7 | 741.81 | 0.62 | 0.12 | −363.46 | 0.44 |
| Age + Group type + Elephants present | 6 | 742.03 | 0.84 | 0.11 | −364.68 | 0.55 |
| Age | 4 | 742.20 | 1.01 | 0.10 | −366.94 | 0.65 |
| Null (intercept only) | 3 | 742.85 | 1.66 | 0.07 | −368.33 | 0.72 |
| Musth status × Age × Group type | 15 | 743.18 | 1.98 | 0.06 | −354.52 | 0.78 |
| Musth status + Age + Group type | 8 | 743.39 | 2.20 | 0.06 | −363.11 | 0.84 |
| Musth status + Age + Group type + Elephants present | 9 | 743.68 | 2.48 | 0.05 | −362.10 | 0.89 |
| Musth status × Age × Group type + Elephants present | 16 | 743.72 | 2.53 | 0.05 | −353.50 | 0.94 |
| Musth status × Age | 9 | 744.58 | 3.39 | 0.03 | −362.55 | 0.97 |
| Musth status + Age | 7 | 744.81 | 3.62 | 0.03 | −364.95 | 0.99 |
| Musth status | 6 | 747.54 | 6.35 | 0.01 | −367.43 | 1.00 |
| **Prosocial behavior: $R^2_c$ = 0.476** | | | | | | |
| Musth status × Age × Group type + Elephants present | 16 | 950.70 | 0.00 | 1.00 | −456.99 | 1.00 |
| Musth status + Age + Group type + Elephants present | 9 | 965.09 | 14.39 | 0.00 | −472.81 | 1.00 |
| Age + Group type + Elephants present | 6 | 969.87 | 19.16 | 0.00 | −478.60 | 1.00 |
| Musth status × Age × Group type | 15 | 971.49 | 20.79 | 0.00 | −468.68 | 1.00 |
| Age × Group type + Elephants present | 7 | 971.70 | 20.99 | 0.00 | −478.40 | 1.00 |
| Musth status × Age | 9 | 983.14 | 32.44 | 0.00 | −481.83 | 1.00 |
| Age | 4 | 983.94 | 33.23 | 0.00 | −487.81 | 1.00 |
| Null (intercept only) | 3 | 984.82 | 34.12 | 0.00 | −489.32 | 1.00 |
| Age + Group type | 5 | 985.52 | 34.81 | 0.00 | −487.52 | 1.00 |
| Musth status + Age | 7 | 985.88 | 35.17 | 0.00 | −485.49 | 1.00 |
| Musth status + Age + Group type | 8 | 986.86 | 36.15 | 0.00 | −484.84 | 1.00 |
| Age × Group type | 6 | 987.56 | 36.85 | 0.00 | −487.44 | 1.00 |
| Musth status | 6 | 989.72 | 39.01 | 0.00 | −488.52 | 1.00 |
| **Dominance behavior: $R^2_c$ = 0.007** | | | | | | |

| | | | | | | |
|---|---|---|---|---|---|---|
| Null (intercept only) | 3 | 787.81 | 0.00 | 0.37 | −390.81 | 0.37 |
| Age | 4 | 789.16 | 1.35 | 0.19 | −390.42 | 0.56 |
| Musth status | 6 | 789.51 | 1.70 | 0.16 | −388.42 | 0.72 |
| Age + Group type | 5 | 791.18 | 3.37 | 0.07 | −390.35 | 0.79 |
| Musth status + Age | 7 | 791.57 | 3.76 | 0.06 | −388.33 | 0.85 |
| Age + Group type + Elephants present | 6 | 792.21 | 4.40 | 0.04 | −389.77 | 0.89 |
| Age × Group type | 6 | 792.46 | 4.65 | 0.04 | −389.89 | 0.93 |
| Age × Group type + Elephants present | 7 | 793.52 | 5.71 | 0.02 | −389.31 | 0.95 |
| Musth status + Age + Group type | 8 | 793.76 | 5.95 | 0.02 | −388.29 | 0.97 |
| Musth status × Age | 9 | 794.02 | 6.21 | 0.02 | −387.27 | 0.98 |
| Musth status + Age + Group type + Elephants present | 9 | 794.16 | 6.35 | 0.02 | −387.34 | 1.00 |
| Musth status × Age × Group type + Elephants present | 16 | 803.69 | 15.88 | 0.00 | −383.48 | 1.00 |
| Musth status × Age × Group type | 15 | 805.00 | 17.19 | 0.00 | −385.43 | 1.00 |
| **Submissive behavior: $R^2_c$ = 0.276** | | | | | | |
| Musth status × Age × Group type + Elephants present* | 16 | 756.07 | 0.00 | 0.52 | −359.67 | 0.52 |
| Musth status × Age × Group type | 15 | 756.51 | 0.44 | 0.42 | −361.19 | 0.94 |
| Age + Group type + Elephants present | 6 | 762.75 | 6.68 | 0.02 | −375.04 | 0.96 |
| Age + Group type | 5 | 762.82 | 6.74 | 0.02 | −376.17 | 0.98 |
| Age × Group type + Elephants present | 7 | 764.14 | 8.07 | 0.01 | −374.62 | 0.99 |
| Age × Group type | 6 | 764.18 | 8.10 | 0.01 | −375.75 | 1.00 |
| Age | 4 | 768.25 | 12.18 | 0.00 | −379.97 | 1.00 |
| Musth status + Age + Group type + Elephants present | 9 | 768.58 | 12.51 | 0.00 | −374.55 | 1.00 |
| Musth status + Age + Group type | 8 | 768.99 | 12.92 | 0.00 | −375.91 | 1.00 |
| Null (intercept only) | 3 | 771.20 | 15.13 | 0.00 | −382.51 | 1.00 |
| Musth status + Age | 7 | 774.09 | 18.02 | 0.00 | −379.59 | 1.00 |
| Musth status | 6 | 776.09 | 20.01 | 0.00 | −381.71 | 1.00 |
| Musth status × Age | 9 | 776.36 | 20.28 | 0.00 | −378.44 | 1.00 |

**Table S3.** Ranked regression models investigating effect of various factors on rates of social behavior in wild elephants. Other statistics include parameterization ($k$), log-likelihood (LL), Akaike's Information Criterion score ($AIC_c$), differences in $AIC_c$ ($\Delta AIC_c$), Akaike weight ($w_i$), and cumulative Akaike weights (cum. $w_i$) calculated with restricted maximum likelihood estimation. Refer to Table S1 for descriptions of each parameter. Asterices (*) indicate term(s) from the best model that were dropped for non-significance in the "best" model; marginal coefficients of determination ($R^2_c$) are given for the best model after non-significant term(s) were dropped.

| **Wild elephants: Rates of social behavior** | ***k*** | **$AIC_c$** | **$\Delta AIC_c$** | **$w_i$** | **LL** | **cum. $w_i$** |
|---|---|---|---|---|---|---|
| **Aggression: $R^2_c$ = 0.371** | | | | | | |
| Age + Group type* + Elephants present* | 9 | 363.73 | 0.00 | 0.52 | −172.15 | 0.52 |
| Age | 7 | 364.92 | 1.18 | 0.29 | −175.02 | 0.80 |
| Age + Group type | 8 | 367.16 | 3.43 | 0.09 | −175.01 | 0.90 |
| Null (intercept only) | 3 | 368.01 | 4.28 | 0.06 | −180.92 | 0.96 |
| Musth status + Age + Group type + Elephants present | 12 | 370.62 | 6.89 | 0.02 | −172.04 | 0.97 |
| Musth status + Age | 10 | 371.80 | 8.07 | 0.01 | −175.02 | 0.98 |
| Age × Group type + Elephants present | 13 | 371.84 | 8.11 | 0.01 | −171.43 | 0.99 |
| Musth status | 6 | 373.60 | 9.86 | 0.00 | −180.47 | 0.99 |
| Musth status + Age + Group type | 11 | 374.15 | 10.42 | 0.00 | −175.01 | 1.00 |
| Age × Group type | 12 | 374.85 | 11.12 | 0.00 | −174.16 | 1.00 |
| Musth status × Age | 13 | 379.00 | 15.27 | 0.00 | −175.01 | 1.00 |
| Musth status × Age × Group type + Elephants present | 21 | 392.48 | 28.75 | 0.00 | −171.19 | 1.00 |
| Musth status × Age × Group type | 20 | 395.53 | 31.80 | 0.00 | −174.11 | 1.00 |
| **Prosocial behavior: $R^2_c$ = 0.239** | | | | | | |
| Age + Group type + Elephants present | 9 | 1078.14 | 0.00 | 0.89 | −529.35 | 0.89 |
| Musth status + Age + Group type + Elephants present | 12 | 1082.99 | 4.85 | 0.08 | −528.22 | 0.96 |
| Age × Group type + Elephants present | 13 | 1084.65 | 6.52 | 0.03 | −527.83 | 1.00 |
| Age + Group type | 8 | 1092.43 | 14.29 | 0.00 | −537.65 | 1.00 |
| Null (intercept only) | 3 | 1094.07 | 15.93 | 0.00 | −543.94 | 1.00 |
| Musth status + Age + Group type | 11 | 1096.87 | 18.73 | 0.00 | −536.37 | 1.00 |
| Musth status | 6 | 1097.50 | 19.36 | 0.00 | −542.42 | 1.00 |
| Age × Group type | 12 | 1098.34 | 20.20 | 0.00 | −535.90 | 1.00 |
| Musth status × Age × Group type + Elephants present | 21 | 1100.92 | 22.79 | 0.00 | −525.41 | 1.00 |
| Age | 7 | 1102.15 | 24.01 | 0.00 | −543.64 | 1.00 |
| Musth status + Age | 10 | 1105.31 | 27.17 | 0.00 | −541.77 | 1.00 |
| Musth status × Age | 13 | 1109.24 | 31.10 | 0.00 | −540.13 | 1.00 |
| Musth status × Age × Group type | 20 | 1113.75 | 35.61 | 0.00 | −533.22 | 1.00 |
| **Dominance behavior: $R^2_c$ = 0.000** | | | | | | |

| | | | | | | |
|---|---|---|---|---|---|---|
| Null (intercept only) | 3 | 812.00 | 0.00 | 0.81 | −402.91 | 0.81 |
| Age | 7 | 817.01 | 5.01 | 0.07 | −401.07 | 0.88 |
| Musth status | 6 | 817.18 | 5.18 | 0.06 | −402.26 | 0.94 |
| Age + Group type | 8 | 819.02 | 7.02 | 0.02 | −400.94 | 0.96 |
| Musth status + Age | 10 | 819.59 | 7.59 | 0.02 | −398.92 | 0.98 |
| Age + Group type + Elephants present | 9 | 821.20 | 9.20 | 0.01 | −400.88 | 0.99 |
| Musth status + Age + Group type | 11 | 821.54 | 9.54 | 0.01 | −398.71 | 0.99 |
| Musth status + Age + Group type + Elephants present | 12 | 823.90 | 11.90 | 0.00 | −398.68 | 1.00 |
| Musth status × Age | 13 | 824.45 | 12.45 | 0.00 | −397.73 | 1.00 |
| Age × Group type | 12 | 825.01 | 13.01 | 0.00 | −399.23 | 1.00 |
| Age × Group type + Elephants present | 13 | 827.39 | 15.39 | 0.00 | −399.20 | 1.00 |
| Musth status × Age × Group type | 20 | 836.00 | 24.00 | 0.00 | −394.35 | 1.00 |
| Musth status × Age × Group type + Elephants present | 21 | 838.71 | 26.71 | 0.00 | −394.30 | 1.00 |
| **Submissive behavior: $R^2_c$ = 0.121** | | | | | | |
| Null (intercept only) | 3 | 743.64 | 0.00 | 0.41 | −368.73 | 0.41 |
| Age + Group type + Elephants present | 9 | 744.99 | 1.35 | 0.21 | −362.78 | 0.62 |
| Age | 7 | 745.04 | 1.40 | 0.20 | −365.08 | 0.82 |
| Age + Group type | 8 | 746.25 | 2.61 | 0.11 | −364.56 | 0.93 |
| Musth status | 6 | 749.50 | 5.86 | 0.02 | −368.42 | 0.95 |
| Age × Group type + Elephants present | 13 | 749.81 | 6.17 | 0.02 | −360.41 | 0.97 |
| Age × Group type | 12 | 750.86 | 7.22 | 0.01 | −362.16 | 0.98 |
| Musth status + Age | 10 | 751.61 | 7.97 | 0.01 | −364.92 | 0.99 |
| Musth status + Age + Group type + Elephants present | 12 | 751.77 | 8.13 | 0.01 | −362.62 | 1.00 |
| Musth status + Age + Group type | 11 | 752.92 | 9.28 | 0.00 | −364.39 | 1.00 |
| Musth status × Age | 13 | 756.25 | 12.61 | 0.00 | −363.63 | 1.00 |
| Musth status × Age × Group type + Elephants present | 21 | 768.85 | 25.21 | 0.00 | −359.37 | 1.00 |
| Musth status × Age × Group type | 20 | 769.39 | 25.75 | 0.00 | −361.04 | 1.00 |

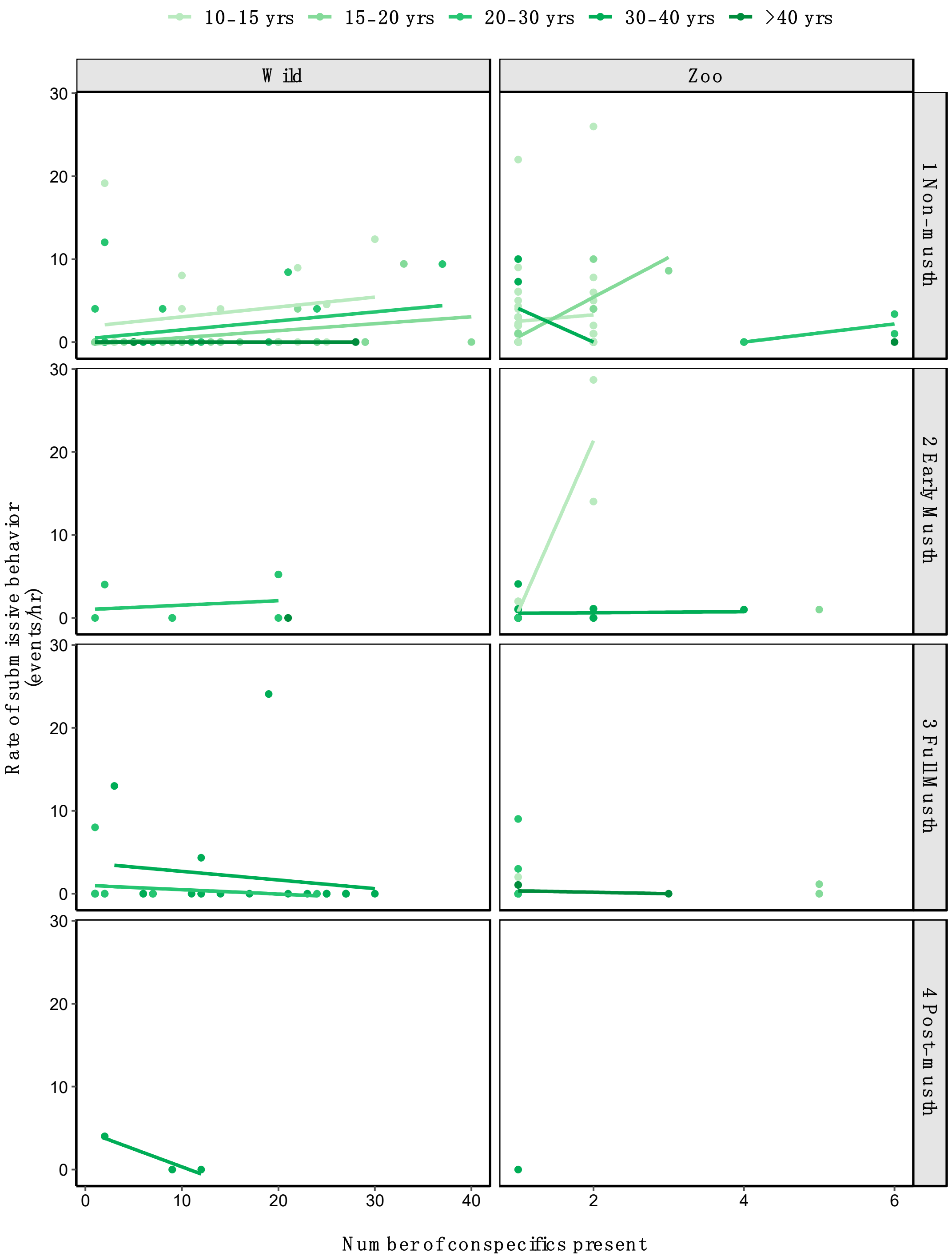


**Figure S2.** Relationship between rates of submissive behavior and the number of conspecifics present during, for wild and zoo-housed male Asian elephants. Closed circles represent individual observation sessions, with regression lines shown for each age class (darker shades represent older age classes). Absence of a regression line for an age class indicates lack of adequate data for construction of a relationship. Note difference in scale on horizontal axis for wild and zoo-housed elephants.
